# Developmental dynamics of myogenesis in the shipworm *Lyrodus pedicellatus* (Mollusca: Bivalvia)

**DOI:** 10.1186/s12983-014-0090-9

**Published:** 2014-12-10

**Authors:** Andrea Wurzinger-Mayer, J Reuben Shipway, Alen Kristof, Thomas Schwaha, Simon M Cragg, Andreas Wanninger

**Affiliations:** Department of Integrative Zoology, Faculty of Life Sciences, University of Vienna, Althanstrasse 14, 1090 Vienna, Austria; Institute of Marine Sciences, University of Portsmouth, Ferry Road, Portsmouth, P04 9LY UK

**Keywords:** Teredinid, Mollusk, Evodevo, Evolution, Ontogeny, Larva, Veliger, Lophotrochozoa

## Abstract

**Background:**

The shipworm *Lyrodus pedicellatus* is a wood-boring bivalve with an unusual vermiform body. Although its larvae are brooded, they retain the general appearance of a typical bivalve veliger-type larva. Here, we describe myogenesis of *L. pedicellatus* revealed by filamentous actin labelling and discuss the data in a comparative framework in order to test for homologous structures that might be part of the bivalve (larval) muscular ground pattern.

**Results:**

Five major muscle systems were identified: a velum retractor, foot retractor, larval retractor, a distinct mantle musculature and an adductor system. For a short period of larval life, an additional ventral larval retractor is present. Early in development, a velum muscle ring and an oral velum musculature emerge. In late stages the lateral and dorsal mantle musculature, paired finger-shaped muscles, an accessory adductor and a pedal plexus are formed. Similar to other bivalve larvae, *L. pedicellatus* exhibits three velum retractor muscles, but in contrast to other species, one of them disappears in early stages of *L. pedicellatus*. The remaining two velum retractors are considerably remodelled during late larval development and are most likely incorporated into the elaborate mantle musculature of the adult.

**Conclusions:**

To our knowledge, this is the first account of any larval retractor system that might contribute to the adult bodyplan of a (conchiferan) mollusk. A comparative analysis shows that a pedal plexus, adductors, a larval velum ring, velum retractors and a ventral larval retractor are commonly found among bivalve larvae, and thus most likely belong to the ground pattern of the bivalve larval musculature.

**Electronic supplementary material:**

The online version of this article (doi:10.1186/s12983-014-0090-9) contains supplementary material, which is available to authorized users.

## Background

With 15.000 extant species, Bivalvia represents the second largest class-level taxon within the Mollusca. Bivalve species are aquatic, laterally compressed lophotrochozoans with two shell valves that are hinged dorsally and interconnected by a ligament, which acts antagonistically against the adductor muscles. The dimyarian arrangement with one anterior and one posterior adductor muscle is characteristic of many bivalves [1]. During development, most marine bivalves undergo a lecithotrophic trochophore stage that is followed by a planktotrophic (sometimes lecithotrophic) veliger larva. Exceptions include the putative basal protobranchs with a pericalymma and certain freshwater bivalves with a parasitic glochidium larva [2,3].

Representatives of the Teredinidae and some Pholadidae (Heterodonta) burrow into and feed on wood and thereby as adults greatly deviate from the typical bivalve anatomy and mode of life. As such, the teredinid *Lyrodus pedicellatus* has an elongated, worm-like body extending well beyond the shell, which acts as a highly specialized drilling tool. The shell surrounds a short, well-developed foot that serves as an attachment organ to the wood and helps facilitate boring [4,5]. The two adductors, typical for heterodonts, are anisomyarian and the smaller anterior adductor is divided into two distinct subportions [1,6]. The valves are interconnected by the adductor muscles and not by the hinge as is the case in other bivalves. The gills not only serve as respiratory and filtering organs, but also harbour bacterial symbionts and house the larvae in brood pouches. While the adult *L. pedicellatus* has a highly derived body, its later-stage larvae bear significant gross morphological resemblance to trochophore and veliger larvae of most other bivalve representatives, despite the fact that *L. pedicellatus* larvae are brooded to the advanced pediveliger or even metamorphically competent stage and have no direct access to the water column (Additional file 1: Figure S1). Thereby, an oval-shaped and densely ciliated retractable velum characterizes the veliger larva of *L. pedicellatus* [7]. During metamorphosis the velum is resorbed [8]. The two convex lobes of the D-shaped (veliger stage) or roundish (pediveliger-stage) larval shell are hinged and cover the soft body of the larva.

**Figure 1:**
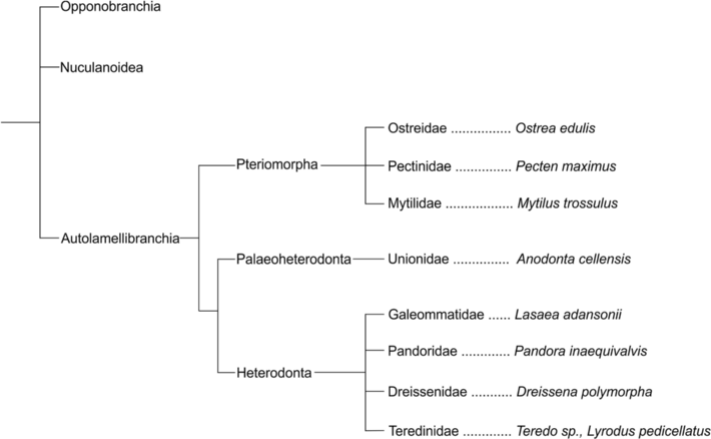
**Suggested relationships of selected bivalve taxa after Giribet** **[** 1 **]** **, with species for which significant data on myogenesis are available specifically mentioned.**

While myogenesis has been described for a number of mollusks (e.g., [9-19]), surprisingly few recent investigations have dealt with bivalve muscle development. Other aspects of bivalve larval anatomy and ontogeny have been addressed by classical light (e.g. [20-23]) as well as electron and confocal laser scanning microscopy (e.g. [24-27]). Herein, we describe myogenesis from early stage larvae to early juveniles in the shipworm *Lyrodus pedicellatus.* The results are compared with the scarce available data on other bivalve taxa (see Figure 1) in order to assess potential homologies and to shed light on the ground pattern of the bivalve (larval) muscular bodyplan.

## Methods

### Animal fixation and F-actin staining

Adult *Lyrodus pedicellatus* were obtained from an infested pier composed of Greenheart wood (*Chlorocardium rodiei*) at Portsmouth, UK. Adults were harvested in June and October 2012 and were dissected in order to yield the larvae. Subsequent cultures were reared in aquaria at the Institute of Marine Sciences, University of Portsmouth. Seawater was taken directly from Langstone Harbour, maintained at a temperature between 15-18°C and a salinity of 36 PSU and kept aerated throughout. Tanks were regularly provided with small panels of wood (pine, measuring 2.5 cm × 2.5 cm × 2.5 cm) for larval settlement.

Larvae were relaxed in 7.5% MgCl_2_, fixed in 4% paraformaldehyde in 0.1M phosphate buffer (PB) and stored in 0.5M PB +0.1% NaN_3_ at 4°C. For decalcification, the larvae were incubated in 0.5M ethylene glycol tetraacetic acid (EGTA) overnight at room temperature. Specimens were then rinsed three times (10 min each) in 0.1M PB. Afterwards, the animals were permeabilized in PBT (0.1M PB +0.2% Triton X-100) overnight at room temperature. F-actin was labelled by fluorescence-coupled phalloidin (Alexa Fluor 488; Molecular Probes, Eugene, OR, USA) in a 1:40 dilution of PBT for 6 to 10 hours in the dark at room temperature. Cell nuclei were stained with DAPI (4′, 6-diamidino-2-phenylindole) (Sigma, St. Louis, MO, USA), followed by three washes in 0.1M PB (10 min each). Stained specimens were mounted on glass slides using Fluoromount G (Southern Biotech, Birmingham, AL, USA).

For details on preparation of the scanning electron micrographs used in Supplemental Figure S1, please see Additional file 2.

### Analysis and digital image processing

A Leica DM 1000 epifluorescence microscope (Leica, Wetzlar, Germany) was used for gross morphological analysis of 318 animals. 64 of them were selected for detailed examinations using a Leica TCS SP5 II confocal laser scanning microscope (CLSM) (Leica, Wetzlar, Germany). Optical sections between 0.2 and 0.5 μm of the whole-mounts were generated and digitally merged into maximum projections. In order to depict the 3-dimensionality of the specimens and to accentuate the regions of interests, the confocal stacks were analyzed with the 3D-reconstruction software Imaris 7.3 (Bitplane, Zürich, Switzerland). Adjustment and optimization of contrast and brightness as well as the conversion into black and white images was performed with Photoshop CS 6 (Adobe Systems, San Jose, CA, USA). Line drawings were generated with Corel Draw X 6 (Corel Corporation, Ottawa, Ontario, Canada).

### Staging of larvae

Staging of specimens based on a timescale referring to oocyte fertilization was not possible due to internal development and brooding in *Lyrodus pedicellatus*. Therefore, size measurements were the prime means of dividing the specimens into individual developmental stages. Besides the developing complexity of the musculature, the formation and loss of morphological features such as the velum and the shell, as well as the degree of calcification, colour and shape of the latter are used herein for defining individual larval stages.

### Orientation of larvae

There exists some confusion concerning axis determination and orientation in bivalve larvae, which largely results from the fact that the morphological dorso-ventral designation used in adult bivalves (whereby ventral marks the axis running from mouth to anus and dorsal the opposite region, often the site of the hinge) has been directly transferred to the orientation in the (pedi-) veliger stages. In postmetamorphic and adult bivalves, the mouth (and foot) usually comes to lie close to the anterior pole, often in the vicinity of an anterior adductor system. Accordingly, the mouth occupies a ventro-anterior position in many postmetamorphic bivalves. In bivalve larvae, the mouth is situated immediately behind the velum, which is shed during metamorphosis. Thus, the apical region, terminating with the apical tuft, is often referred to as ventral in bivalve larvae (e.g., [23]; see also [28]). This, however, is in stark contrast to axis determination used in almost all other lophotrochozoan larvae, where the apical tuft marks anterior and the opposite pole posterior. In order to conform to this terminology widely used in comparative lophotrochozoan larval anatomy, we use the latter designation herein (apical tuft = anterior, opposite of apical tuft = posterior, position of the foot = ventral), although we are aware that this may not correspond to the condition exhibited in adult (postmetamorphic) bivalves.

## Results

**Figure 2:**
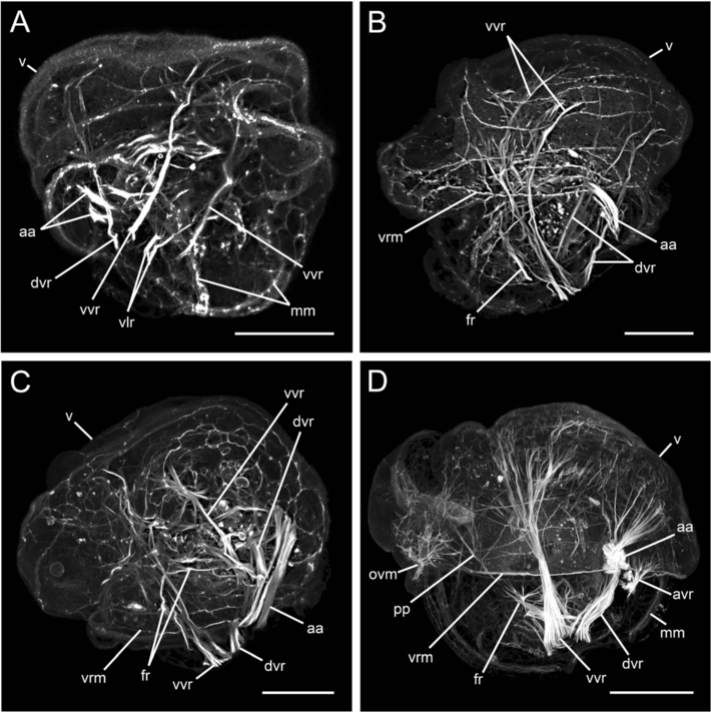
**Confocal micrographs showing myogenesis in four subsequent early larval phases of** ***Lyrodus pedicellatus*** **.** Scale bars represent 20 μm, except in **D**, in which it equals 50 μm. All aspects are in lateral view, **C** is slightly tilted in anterodorsal direction. Anterior is upwards in all aspects. Ventral is to the left, except in **A**, where it is to the right. **(A)** Earliest veliger stage investigated, showing two muscle bundles of the paired ventral larval retractors (vlr) and a pair of ventral (vvr) and dorsal velum retractors (dvr), which extend into the velum (v). The anterior adductor muscle (aa), divided into two bundles, is visible in the dorsal position of the larva and the mantle musculature (mm) surrounds the mantle margin. **(B)** Early veliger with the velum ring musculature (vrm) and first muscle fibres of the paired foot retractor (fr). Note that the pair of ventral velum retractors now each consists of two parallel muscle bundles. **(C)** Marginally more advanced veliger than the specimen shown in B with fibres of the left and right foot retractor fusing in the median plane. **(D)** Mid-veliger with accessory velum retractor (avr) that is attached to the outer surface of the anterior adductor and spreads into the velum. The foot retractor elongates along the median plane. At the ventral side of the velum, a muscle grid (oral velum musculature; ovm) that has formed contact to the velum ring musculature and to the first fibers of the pedal plexus (pp) develops in the postoral region. Note the distinct separation of the pedal plexus and the foot retractors.

**Figure 3:**
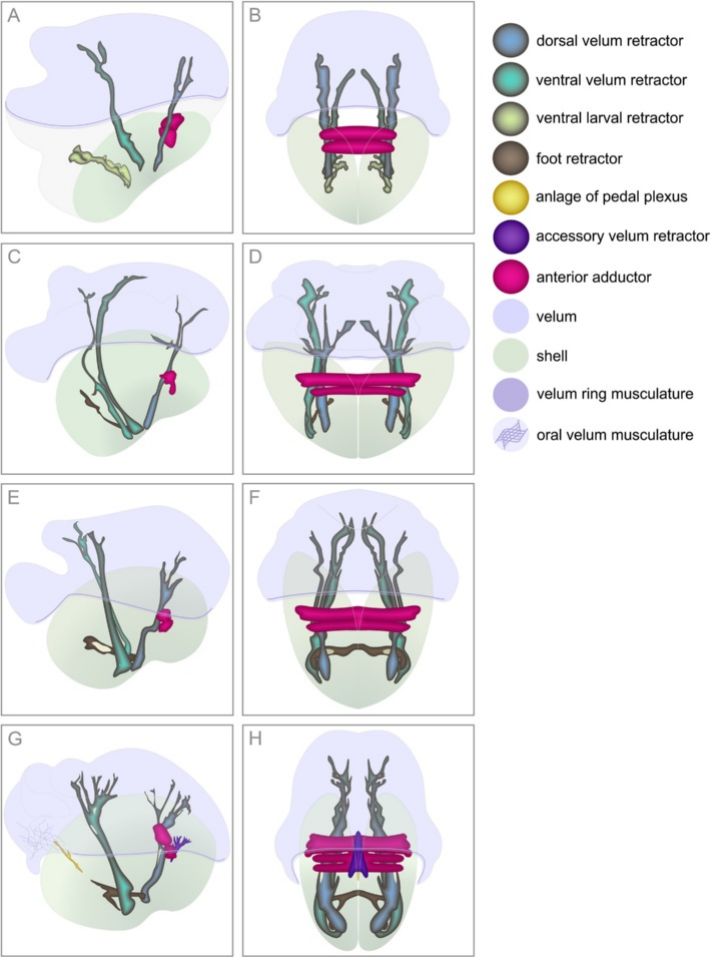
**Graphic representation based on 3D-reconstructions of larval myogenesis in** ***Lyrodus pedicellatus*** **.** Anterior is upwards in all aspects. **A**, **C**, **E**, **G** is in a lateral view and ventral is to the left. **B**, **D**, **F**, **H** is in dorsal view. **(A)** Earliest veliger stage investigated with oval-shaped valves and velum with a delicate velum muscle ring. Ventral and dorsal velum retractors extend into the velum. Ventral larval retractors project ventrally. The anterior adductor muscle has contact to the dorsal velum retractor. **(B)** Earliest veliger stage investigated showing the two portions of the anterior adductor muscle which spans between the left and right valve. Paired velum retractors project into the velum. **(C)** Early veliger larva with the anlage of the foot retractors that originate at the paired ventral velum retractors, which now consist of two distinct bundles. Note that the ventral larval retractor has already disintegrated in this early stage. **(D)** Early veliger larva with the developing foot retractors which project towards the midline. **(E)** Slightly further developed veliger showing two intercrossing bundles of the ventral velum retractors. **(F)** Veliger with the left and right foot retractor that meet in the midline and fuse. **(G)** Developing median muscle fibres that form the anlage of the pedal plexus in the mid-veliger larva. An oral velum musculature appears. On both sides, the two branches of the ventral velum retractor fuse. Note that the accessory velum retractor is attached to the outer surface of the anterior adductor muscle. **(H)** Mid-veliger with unpaired accessory velum retractor.

The youngest developmental stage investigated was characterized by the lack of a velum or a shell, was less than 60 μm in length and did not show any muscle-specific signal. In the earliest veliger stage investigated (Figure 2A), ellipsoid shells are formed. They are interconnected in the posterior region of the larva and envelope not more than one third of the body (Figure 3A). Underneath the cap-like velum a delicate velum muscle ring is present (Figure 3A, B). In addition, several well-developed muscle bundles have formed at this stage. The anterior adductor muscle spans between the left and the right valve in the dorsal part of the shell and is divided into two bundles (Figures 2A and 3B). A pair of dorsal velum retractors attaches to the area of the developing shell, extends into the velum and runs along the inner surface of the anterior adductor (Figures 2A and 3A, B). Clearly separated from the dorsal velum retractor, a pair of ventral velum retractors appears and extends toward the central portion of the velum (Figures 2A and 3A). Ventrally, clearly distinct from the ventral velum retractor, a ventral larval retractor, which consists of two bundles, develops and projects toward the anterior part of the velum (Figures 2A and 3A).

In early veliger larvae, the ventral larval retractor disappears (Figures 2B and 3C, D). The dorsal velum retractors elongate and few fibres project into the velum, where the velum ring musculature is now fully developed and thicker than in the previous stage (Figures 2B and 3C, D). The ventral velum retractors now consist of two distinct, parallel bundles, which extend far into the velum (Figures 2B and 3C). Delicate muscle fibres, which originate at the base of the left and right dorsal branches of the ventral velum retractors and at the lower third of both ventral branches, represent the anlage of the foot retractors, which project toward the midline (Figures 2B and 3C, D). The anterior adductor expands between the shell valves, which now reach the velum and are rounded in shape (Figure 3C, D).

In slightly older veligers, the shell has a distinct concave indentation in the posterior region near the attachment area of the velum retractors (Figure 3E). In this stage, the two bundles of the ventral velum retractors intercross (Figures 2C and 3E). The left and right muscle bundles, which later will develop into the foot retractors, fuse in the median plane (Figures 2C and 3F). The anterior adductor increases in size and the velum ring musculature still underlies the edge of the velum (Figures 2C and 3E, F).

In mid-veligers, the oral part of the velum ring disintegrates, while its remaining fibres connect to the oral velum musculature, which consists of a muscle grid that appears to interconnect postoral soft tissues (Figures 2D and 3G). The base of the foot retractors splits and forms a second ventral attachment zone, close to the ventral velum retractor (Figure 3G, H). Along the median plane the foot retractor elongates and adjacent to it, muscle fibres appear and connect to the oral velum musculature (Figures 2D and 3G). The velum retractors increase in size and fan out into the velum. The two portions of the ventral velum retractors fuse but maintain different shapes (Figures 2D and 3G). An unpaired accessory velum retractor appears in close proximity to the well-defined anterior adductor (Figures 2D and 3G, H). The shell becomes more round and grows anteriorly (Figure 3G, H).

**Figure 4:**
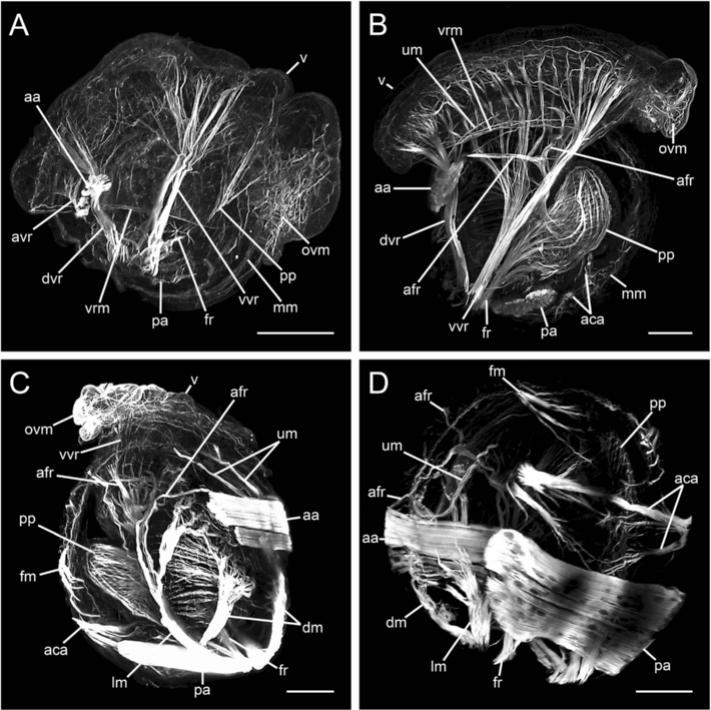
**Myogenesis from late veliger to the early juvenile in** **Lyrodus pedicellatus (confocal micrographs)** **.** Scale bars: 50 μm. **A**, **B**: Lateral view. **C**: Dorso-lateral view. **D**: Ventro-lateral view. Anterior up and ventral right, except for **C**, where ventral is left. **(A)** Late veliger with paired dorsal (dvr) and ventral velum retractors (vvr). An accessory velum retractor (avr) is attached to the anterior adductor (aa). The pedal plexus (pp) contacts the oral velum musculature (ovm). The pedal plexus projects toward the foot retractor (fr). The posterior adductor (pa) has developed ventrally of the ventral velum retractor. Compact fibres indicate the margin of the mantle musculature (mm). **(B)** Mid-pediveliger. The velum (v) becomes smaller. The velum ring musculature (vrm) loses its solid appearance. The accessory velum retractor (avr) disappears, the dorsal (dvr) and ventral velum retractor (vvr) are predominant. An accessory adductor (aca) has developed. Pedal plexus and foot retractor have fused and form the foot musculature. The accessory foot retractors (afr) divide halfway into two branches. A diffuse mantle musculature (mm) and a U-shaped mantle musculature (um) originate at the anterior edge of the anterior adductor. **(C)** Late pediveliger showing disintegration of larval structures. The velum (v) shrinks, the oral velum musculature (ovm) is pushed together, the velum ring musculature disappears. The U-shaped mantle musculature (um), connected to the anterior adductor (aa), elongates. The velum retractors have largely disintegrated, except for one bundle of the ventral velum retractor (vvr). Lateral (lm) and dorsal mantle musculature (dm) lie close to the shell. Foot retractors (fr), accessory foot retractors (afr) and pedal plexus (pp) form the foot musculature. Posterior adductor (pa) and accessory adductor (aca) are well-developed. A finger-shaped mantle musculature (fm) is formed. **(D)** Early juvenile with velum lost. U-shaped mantle musculature (um) fuses with finger-shaped mantle musculature (fm).

**Figure 5:**
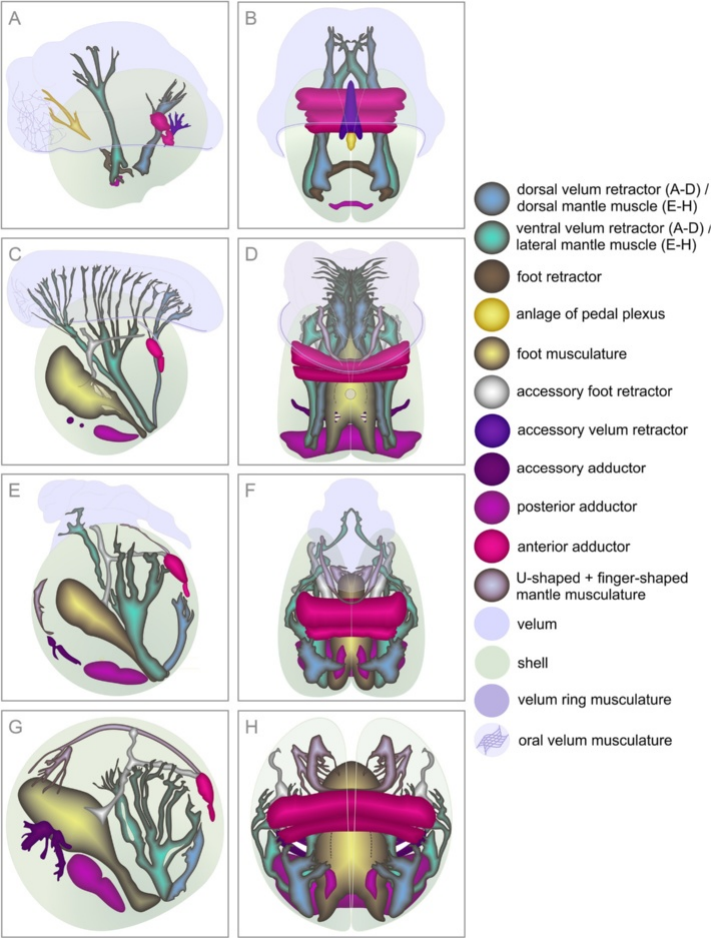
**Graphic representation based on 3D-reconstructions of larval myogenesis in** ***Lyrodus pedicellatus*** **. A**, **C**, **E**, **G** are in lateral view, anterior is upwards, ventral to the left. **B**, **D**, **F**, **H** are in dorsal view, anterior is upwards. **(A)** Late veliger with round shells and a fully developed velum. The foot consists of two separated portions, the foot retractor and the anlage of the pedal plexus. **(B)** Late veliger with developing posterior adductor muscle which extends between the left and the right valve. **(C)** Mid-pediveliger with shrinking velum. The foot retractors and the pedal plexus have fused and together with a two-pronged accessory foot retractor form the foot musculature. The paired velum retractors are fully developed. An accessory adductor muscle appears. **(D)** Mid-pediveliger with a solid anterior and posterior adductor muscle. The accessory adductor muscle spans between the valves. Note the developing U-shaped mantle musculature. The accessory velum retractor has disappeared. **(E)** Late pediveliger with a largely atrophied velum. The velum ring musculature has disintegrated. For the most part, the velum retractors are resorbed distally. Paired finger-shaped mantle musculature develops. **(F)** Late pediveliger with remaining basal parts of the velum retractors which transform into the lateral and dorsal mantle musculature. This paired mantle musculature gains volume and has shifted more laterally compared to previous stages. **(G)** Early juvenile with shed velum. Ventrally, the U-shaped mantle musculature forms contact to the finger-shaped mantle musculature. **(H)** Early juvenile with lateral and dorsal mantle musculature at the periphery, parallel to the inner surface of the spherical shell.

In the late veliger stage, the dorsal and ventral pair of the velum retractors are both pronounced and numerous distal branches project into the velum. The unpaired accessory velum retractor is distinct and the anterior adductor is well-developed (Figures 4A and 5A, B). The posterior adductor forms ventrally from the insertion area of the ventral velum retractor and attaches to both valves (Figures 4A and 5B). The muscle fibres that follow the trajectory of the foot retractors become thicker and form three strong branches along the median plane. These are the anlage of the pedal plexus, which at this stage has no contact to the foot retractor (Figures 4A and 5A, B). Distally, at its ventral side, the pedal plexus is still connected to the muscle grid in the velum that now reaches its largest dimension. When expanded, it overhangs two-thirds of the shell (Figure 5A).

In the mid-pediveliger, the velum shrinks in the oral region such that the remaining parts of the oral velum musculature become denser and lie immediately in front of the tips of the ventral velum retractor (Figures 4B and 5C). The velum ring musculature starts to disintegrate from the ventral side (Figures 4B and 5C). Both the dorsal and the ventral velum retractor are remarkably well pronounced at this stage (Additional file 1: Figure S1B). Their main trunks split into several delicate bundles, which project toward the median plane from the left and the right. They enter the velum, where the branches fan out (Figures 4B and 5C, D). The dorsal velum retractor maintains close contact to the anterior adductor, to which a small muscle band is attached anteriorly (Figures 4B and 5C, D). The band runs along the edge of the left and right shell and elongates as a U-shaped mantle musculature toward the ventral side (Figures 4B and 5D). The accessory velum retractor disappears. The posterior adductor, which lies close to the inner surface of the shell, grows and surpasses the size of the anterior adductor (Figures 4B and 5C, D). Anterior to the posterior adductor a solid two-pronged accessory adductor reaches between the valves (Figures 4B and 5C, D). The pedal plexus fuses with the foot retractor and a complex muscular grid develops (Figures 4B and 5C, Additional file 1: Figure S1B). The two insertion sites of the foot retractor are fused at their base (Figure 5C). At this point, the foot retractor is clearly separated from the ventral velum retractor (Figure 5D, Additional file 1: Figure S1B). An accessory foot retractor develops (Additional file 1: Figure S1B) on the left and right side of the lateral area of the pedal plexus, runs parallel to the inner surface of the shell and furcates halfway into two branches on either side. One end projects toward the anterior edge of the anterior adductor, the other attaches to the shell (Figures 4B and 5C). The convex indentation in the posterior area of the shell flattens and the outline of the valves is almost round (Figure 5C).

In late pediveliger stages, the velum shrinks from the aboral side, degenerates further and the velum ring musculature disappears (Figures 4C and 5E). The dorsal velum retractors no longer have contact to the velum and distally become entirely resorbed (Figures 4C and 5E, F). Conversely, the basal part now appears more solid and becomes incorporated into the mantle as dorsal mantle musculature (Figures 4C and 5E, F). For the most part, the ventral velum retractor is no longer connected to the velum (Figures 4C and 5E). Only a single branch reaches the central part of the velum (Figures 4C and 5E, F). Similar to the dorsal velum retractor, the stem of the ventral velum retractor becomes thicker and develops into the well-pronounced lateral mantle musculature (Figures 4C and 5E, F). Due to its atrophying distal parts, the dorsal velum retractor loses the contact to the anterior adductor. Especially the anterior bundle of the anterior adductor increases in diameter (Figures 4C and 5E, F). The posterior adductor is massive and the accessory adductor is well-developed (Figures 4C and 5E). The accessory foot retractor connects to the anterolateral edge of the anterior adductor (Figures 4C and 5E). Ventral to the tip of the foot, a pair of finger-shaped mantle musculature develops near the inner rim of the left and right shell and the U-shaped mantle musculature extends along the shell margin toward the ventral side (Figures 4C and 5E, F).

In the last developmental stage analyzed, the velum is shed and the branches of the velum retractors are lost (Figures 4D and 5G, H). The shell is round and the animal now appears spherical (Figures 4D and 5G, H). The anterior and the posterior adductors are massive, the accessory adductor is pronounced and the foot is predominant with compact retractors (Figures 4D and 5G, H). The U-shaped mantle musculature, which extends from the anterior surface of the anterior adductor, forms contact with the paired ventral finger-shaped mantle musculature (Figures 4D and 5G). A solid, lateral mantle musculature on both sides of the inner shell surface originates dorsal of the foot and fuses with the base of the dorsal mantle musculature (Figures 4D and 5G). After a short distance it divides into a solid dorsal and ventral branch, whereby the latter branch again subdivides into three parts, which again split and terminate in the region of the accessory foot retractor (Figures 4D and 5G, H). All branches of the lateral and dorsal mantle musculature lie laterally at the periphery (Figures 4D and 5H), except for the remnant of the single branch of the ventral velum retractor, which maintains the straight position next to the median plane (Figure 5H).

The mantle musculature originates alongside the mantle margin. Diffuse mantle muscle fibres appear all over the mantle and in later stages the fibres exhibit a more or less regular pattern in some areas (Figures 2D and 4A, B).

**Figure 6:**
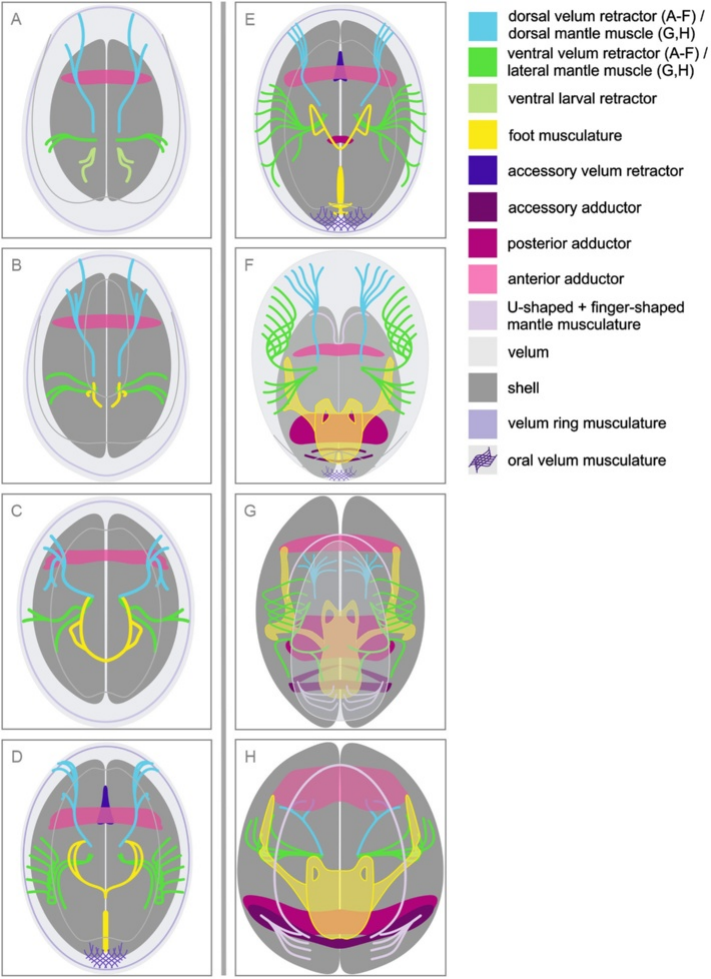
**Schematic representations of myogenesis from the early veliger to the early juvenile in** ***Lyrodus pedicellatus*** **.** Anterior view, apical faces the viewer and dorsal is upwards in all aspects. Folds of the velum are displayed by grey lines. **(A)** Very early veliger with paired dorsal and ventral velum retractors. Note that the paired ventral larval retractor only exists in this early stage. The anterior adductor muscle spans between the valves. **(B)** Early veliger showing paired anlagen of the foot retractor as a first component of the foot musculature. **(C)** Marginally older veliger with fused left and right foot retractors in the middle. **(D)** Mid-veliger with median muscle fibres in the longitudinal projection of the foot retractor. The unpaired accessory velum retractor is attached to the anterior adductor muscle. An oral velum musculature appears. **(E)** Late veliger larva. The median muscle fibres of the foot increase in size, manifesting as pedal plexus which is not yet in contact with the foot retractors. The posterior adductor muscle develops. **(F)** Mid-pediveliger with accessory adductor muscle that reaches between the valves. The pedal plexus unites with the foot retractors and together they form the foot. The paired accessory foot retractors have formed on both sides. The U-shaped mantle musculature forms and extends ventrally. The accessory velum retractor disappears. **(G)** Late pediveliger with reduced velum. Note the finger-shaped mantle musculature. The accessory foot retractor forms contact to the anterior adductor muscle on both sides. **(H)** Early juvenile. Note that the velum has been shed. Partially atrophied velum retractors have transformed into lateral and dorsal mantle muscles. Ventrally, the U-shaped mantle musculature connects to the finger-shaped mantle musculature.

Taken together, the data presented herein show that early-stage *Lyrodus pedicellatus* veliger larvae, with a length of less than 60 μm, have already developed the anlagen of all major larval muscles (Figure 6A, Table 1). The ventral larval retractor disintegrates in slightly older larvae (Figure 6B, Table 1). The foot retractors, which originate at the base of the larval retractors, start to develop in the early veliger larva (Figure 6B, Table 1). The formation of the foot is successively elaborated (Figure 6B-H, Table 1). The velum retractors continuously expand with some portions being subsequently resorbed while others are transformed into mantle musculature (Figure 6, Table 1).

**Table 1:** **Larval myogenesis in** ***Lyrodus pedicellatus***

**Muscles**		**Stage 1**	**Stage 2**	**Stage 3**	**Stage 4**	**Stage 5**	**Stage 6**	**Stage 7**	**Stage 8**
Ventral larval retractor		**+**	**-**	**-**	**-**	**-**	**-**	**-**	**-**
Velum ring musculature		**+**	**+**	**+**	**+**	**+**	**+ / -**	**-**	**-**
Dorsal velum retractor		**+**	**+**	**+**	**+**	**+**	**+**	**-**	**-**
Ventral velum retractor		**+**	**+**	**+**	**+**	**+**	**+**	**+ / -**	**-**
Anterior adductor		**+**	**+**	**+**	**+**	**+**	**+**	**+**	**+**
Foot retractor	anlage + → median fusion ++ → prolongation +++ → fusion with pedal plexus and accessory foot retractor ++++	**-**	**+**	**++**	**+++**	**+++**	**++++**	**++++**	**++++**
Pedal plexus	anlage + → expansion ++ → fusion with foot retractor and accessory foot retractor +++	**-**	**-**	**-**	**+**	**++**	**+++**	**+++**	**+++**
Oral velum musculature		**-**	**-**	**-**	**+**	**+**	**+ / -**	**-**	**-**
Accessory velum retractor		**-**	**-**	**-**	**+**	**+**	**-**	**-**	**-**
Posterior adductor		**-**	**-**	**-**	**-**	**+**	**+**	**+**	**+**
Accessory foot retractor		**-**	**-**	**-**	**-**	**-**	**+**	**+**	**+**
Accessory adductor		**-**	**-**	**-**	**-**	**-**	**+**	**+**	**+**
U-shaped mantle musculature		**-**	**-**	**-**	**-**	**-**	**+**	**+**	**+**
Finger-shaped mantle musculature		**-**	**-**	**-**	**-**	**-**	**-**	**+**	**+**
Dorsal mantle musculature		**-**	**-**	**-**	**-**	**-**	**-**	**+**	**+**
Lateral mantle musculature		**-**	**-**	**-**	**-**	**-**	**-**	**- / +**	**+**

character: (**+**) present; (**-**) absent; (**+/-**) decreasing; (**-/+**) increasing.

stage **1** = earliest veliger investigated; stage **2** = early veliger; stage **3** = mid-veliger 1; stage **4** = mid-veliger 2;

stage **5** = late veliger; stage **6 =** mid-pediveliger; stage **7** = late pediveliger; stage **8** = early juvenile.

## Discussion

The analysis of myogenesis provided herein is based on an unprecedented sequence of bivalve larval stages from early veliger to early juveniles. Five major muscle systems, which consist of altogether 17 distinct subunits, were identified and their genesis including their disintegration or transformation are documented and in the following compared to other bivalve and molluscan representatives.

### Comparative analysis of myogenesis in Bivalvia

#### Adductor systems

In *Lyrodus pedicellatus*, the adult dimyarian arrangement with an anterior and a posterior adductor muscle is already established during larval development. Interestingly, the pteriomorphan *Ostrea edulis* has a monomyarian arrangement with only one adductor muscle as an adult, but during the larval phase, two adductor muscles form, of which the anterior one degenerates during metamorphosis [23]. In the glochidium larva of the freshwater mussel *Anodonta cellensis* (Palaeoheterodonta), the larval adductor completely disintegrates during the parasitic phase and the adult adductors form *de novo* [22] (Table 2). In *L. pedicellatus*, the anterior adductor is formed early in larval development, and at that stage is the only muscle capable of closing the shell (Table 1). It is divided into two distinct parts, similar to the pteriomorph *Pecten maximus* and the heterodont *Dreissena polymorpha* [21,24,29]. The anterior adductor in *L. pedicellatus* remains smaller than the posterior adductor. While the posterior adductor is a single muscle bundle in scallop larvae, it splits into two parts in *O. edulis*, and is divided into several bundles in the heterodont *D. polymorpha* ([21,23,29]). In *L. pedicellatus* the posterior adductor muscle appears to be divided into multiple units. Our study provides the first account of an accessory adductor for any larval bivalve and may constitute an apomorphy of either *L. pedicellatus*, the genus *Lyrodus*, or even a higher taxonomic unit (Table 2). Although the accessory adductor develops late in the larval phase (Table 1), it is nevertheless well-developed and of complex structure by the end of this stage.

**Table 2:** **Muscle systems in larvae of 9 bivalve species**

***Species*** **family**	**Foot musculature: no. of pairs, except protractor (unpaired)**			**Mantle musculature: no. of pairs**				**Adductor unpaired**			**Velum retractor no. of pairs, except accessory retractor (unpaired)**				**Oral velum musculature unpaired**	**Velum ring musculature**	**Ventral larval retractor no. of pairs**
	**Foot retractor**	**Access. foot retractor**	**Protractor**	**U-shaped**	**Finger-shaped**	**Lateral**	**Dorsal**	**Anterior**	**Posterior**	**Other**	**Dorsal**	**Ventral**	**Accessory**	**Total number described**			
*Ostrea edulis* Ostreidae	1	1	?	?	?	?	?	1	1	?	1	1	?	3	?	?	?
*Pecten maximus* Pectinidae	?	1	?	?	?	?	?	1	1	?	?	?	?	4	?	-	3
*Mytilus trossulus* Mytilidae	?	?	?	1	?	?	?	1	1	?	?	?	?	3	?	1	3
*Anodonta cellensis* Unionidae	1	1	?	?	?	?	?	1	1	1 (larval)	-	-	-	-	?	-	-
*Lasaea adansonii* Galeommatidae	1	1	1	1	?	?	?	1	1	?	?	?	?	3	?	?	?
*Pandora inaequivalvis* Pandoridae	1	1	?	?	?	?	?	1	1	?	1	1	?	3	?	?	?
*Dreissena polymorpha* Dreissenidae	1	?	?	?	?	?	?	1	1	?	1	1	-	2	?	?	1
*Teredo sp.* Teredinidae	?	1	?	?	?	?	?	?	?	?	1	1	-	2	?	?	-
*Lyrodus pedicellatus* Teredinidae	1	1	-	1	1	1	1	1	1	1 (accessory)	1	1	1	3	+	+	1

character: (**+**) present; (**-**) absent; (**?**) unknown.

#### Foot retractor system and pedal plexus

The massive foot retractors in the larva of *Lyrodus pedicellatus* attach in a posterior area near the hinge and, due to their similar relative position in the larval body, are considered homologous to the posterior retractors of the pteriomorphans *Ostrea edulis* and *Mytilus edulis,* the heterodonts *Lasaea adansonii* and *Pandora inaequivalvis* and the palaeoheterodont *Anodonta cellensis* (the foot retractors in *O. edulis* have previously been described as “cruciform retractor” because their medial bundles cross the median body plane) ([22,23,25,30-32]; Table 2). Likewise for positional reasons, the paired accessory foot retractor in *L. pedicellatus* most likely corresponds to the “anterior retractor” described for *O. edulis, M. edulis, L. adansonii, P. inaequivalvis* and *A. cellensis* [22,25,30-32] (Table 2), although most studies do not provide enough details to unequivocally settle this issue. In *Pecten maximus* unidentified fibres, which emerge from either end of the anterior bundle of the anterior adductor, extend along the bodywall lining [24] and, according to their position, they are likely homologous to the accessory foot retractor of *L. pedicellatus*. In *Teredo*, another common shipworm, a coniform hump extends and shows the same growth direction as the accessory foot retractor of *L. pedicellatus* [20] (Table 2). In *Dreissena polymorpha*, the foot retractors have been described as the last muscles to form in the larva [21]. This is in sharp contrast to *L. pedicellatus,* where the first muscle fibres of the foot retractors branch off the velum retractors in an early larval stage (Table 1). Also in scallop larvae, foot retractors probably originate from velum retractors [29].

A single protractor, present in *Lasaea adansonii* [25], was not found in *Lyrodus pedicellatus* (Table 2). In general, foot development in *L. pedicellatus* starts early in larval life and is successively refined, with the foot musculature reaching its sophisticated architecture at the end of the larval phase (Table 1).

#### Velum ring musculature

A muscle ring underneath the developing velum is already present in the earliest *Lyrodus pedicellatus* veliger stage investigated and disintegrates in the late pediveliger stage (Table 1). This muscle ring has no contact to the velum retractors, whereas in an unidentified autobranch bivalve the velum retractors have been shown to insert at the velum muscle ring [33]. The putative homologous prototroch muscle ring in *Mytilus trossulus* has been described as being transformed into larval retractors during the veliger stage, but this surprising finding calls for more detailed assessment ([26]; Table 2).

#### Larval retractor systems

The bivalve larval muscle systems that so far have been described in most detail are the retractors and protractors. The results obtained are as diverse as the species examined. Whereas retractors and protractors are striated in *Mytilus trossulus*, there is no evidence for a striation in *Lyrodus pedicellatus*, although transmission electron microscopy would be needed to unequivocally settle this issue (cf. [26] and data herein). Similar to *Teredo* [20], *Lyrodus pedicellatus* has two pairs of velum retractors from the onset of myogenesis, whereas the unpaired accessory velum retractor, only found in *L. pedicellatus,* is added later in development and disappears shortly afterwards (Tables 1 and 2). *Pecten maximus* and probably *Tridacna maxima*, *Tridacna squamosa* and *Tridacna crocea* have four pairs, while *Lasaea adansonii* has three larval retractors, despite its almost direct development and the lack of a velum [24,25,34,35]. Three pairs of velum retractors are also found in *Mytilus trossulus, Ostrea edulis, Pandora inaequivalvis* and *Dreissena polymorpha* [21,23,26,30]. The ventral pair in *D. polymorpha*, adjacent to the anus, is presumably homologous to one of the three posterior retractor pairs in *P. maximus*, which likewise attach near the anus [24]. Due to their position, the three posterior protractors in *M. trossulus* [26] may be homologous to both above-mentioned muscles and are therefore considered as ventral larval retractors herein (Table 2). While the ventral retractor in *D. polymorpha* is maintained until the late veliger stage and the protractors in *M. trossulus* and the posterior retractors in *P. maximus* are present until the pediveliger stages, the ventral larval retractor of *L. pedicellatus,* which grows in the same direction*,* only exists in a very early veliger stage ([21,24,26]; Table 1). In contrast to 3-4 pairs of velum retractors and diverse larval retractors in pteriomorphans, the heterodont *L. pedicellatus* eventually displays two pairs of velum retractors and one pair of short-lived ventral larval retractors. While the distal parts of the ventral and dorsal velum retractors become successively atrophied and eventually degenerate, the basal portion doubles in volume and develops into the future lateral and dorsal mantle musculature, thereby maintaining their distinct insertion sites at the shell.

#### Mantle musculature

As in *Mytilus trossulus* and *Lasaea adansonii*, the mantle edge in *Lyrodus pedicellatus* is held in position partially by a U-shaped mantle musculature ([25,26]; Table 2). In addition, lateral and dorsal mantle muscle bundles are present in *L. pedicellatus*. The finger-shaped mantle musculature on the ventral side has not been described for any other bivalve so far and may constitute a teredinid apomorphy, which may be constrained by the highly derived overall bodyplan of a wood-boring shipworm (Table 2). However, since detailed comparative studies using modern methods are still largely lacking for these enigmatic bivalves, this cannot be clarified at present.

### Comparison of the prototroch muscle ring and larval retractors among mollusks

*Lyrodus pedicellatus* larvae display a muscle ring that underlies the prototroch and, in later stages, the velum. Such a muscle is also known from larval Gastropoda [11,12,17,18], Polyplacophora [13], Neomeniomorpha [36] and Chaetodermomorpha [16], but not Scaphopoda [14] (the monoplacophoran condition remains unknown). This velum ring is usually connected to the main larval retractor in gastropods [17,18]. In addition, in the vetigastropod *Haliotis rufescens,* the caenogastropod *Polinices lewisii,* the patellogastropod *Patella caerulea,* the opisthobranch *Aplysia californica* and the nudibranch *Aeolidiella stephanieae*, the larval retractors do form connections to adjacent regions such as the foot musculature, in addition to their main insertion sites within the velar lobes [10-12,17,18,37]. The velum retractors of *L. pedicellatus* neither have contact with the velum ring musculature, nor do they insert at other muscle systems, but instead fan out into the velar tissue.

Crucial differences are also obvious with respect to the timing of disintegration of the larval retractors in the various conchiferan taxa. In the gastropods *Haliotis kamtschatkana, Patella caerulea, Aeolidiella stephanieae* and *Nerita melanotragus*, the larval retractors are resorbed during metamorphosis [9,11,18,19]. However, in the caenogastropod *Polonices lewisii,* resorption of the larval retractor muscle already begins soon after hatching [10]. The adult muscles arise independently from the larval shell muscles in all bivalves and gastropods investigated so far. Although the future fate of the dorsal and ventral mantle musculature is unknown for *Lyrodus pedicellatus*, their remodelling into shell-inserted mantle retractors during late larval life may indicate that they are retained in the adult shipworm. If true, the former assumption that the conchiferan larval shell musculature is generally independent from that of the adult [38], would have to be reconsidered at least for bivalves. Overall, *L. pedicellatus* is a prime example that larval shell muscles may be remodelled and subsequently integrated into the postmetamorphic bodyplan, thus raising the question as to what extent this may also be the case in other conchiferan mollusks.

## Conclusion

The early degeneration of the velum, the ventral larval retractor and the smaller number of velum retractors reflect, compared to other planktotrophic bivalve clades, a trend toward an abbreviated larval phase in *Lyrodus pedicellatus*. In contrast to gastropods, at least certain portions of the larval retractor muscles of *Lyrodus pedicellatus* most likely do contribute to the postmetamorphic muscular bodyplan. The foot retractor originates in very close proximity to the larval velum retractor and the lateral and dorsal mantle musculature constitutes a remodelled derivative of the larval velum retractors in early juveniles. To what extent, if at all, these muscles are incorporated into the definite adult, worm-like body of *Lyrodus pedicellatus* may only be assessed by analysis of advanced juveniles, which were not available for the present study. This is also true for the fate of other larval muscles including the accessory adductor and the finger-shaped mantle musculature, which so far are only known from *L. pedicellatus.*

Although only few data on bivalve myogenesis are currently available, certain muscular subsets appear to be conserved among the larvae of the various bivalve subtaxa and thus were most likely part of the larval bivalve groundplan. These include a dense pedal plexus, distinct sets of adductors as well as various velum retractors. The larval velum ring was found in *Lyrodus* and *Mytilus* and its presence in most other molluskan taxa confirms homology of this system among bivalves and the entire Mollusca. The presence of distinct prototroch/velum retractors in Bivalvia and Gastropoda (but not Scaphopoda) and their absence in Aculifera suggests co-evolution of these systems with embryonic and/or larval shell(s), either in these two clades alone or at the base of Conchifera (with secondary loss in scaphopods and cephalopods).

## Additional file

Additional file 1: Figure S1. Scanning electron micrographs of late-stage *Lyrodus pedicellatus* larvae. Additional file 2:
**Description of the methods used to generate the scanning electron micrograph shown in Supplemental Figure 1.**
