# Supplementary figures and images for: Developmental dynamics of myogenesis in the shipworm Lyrodus pedicellatus (Mollusca: Bivalvia)
Source: Front Zool. 2014 Dec 10;11:90. doi: 10.1186/s12983-014-0090-9 (PMC4282732; doi:10.1186/s12983-014-0090-9)

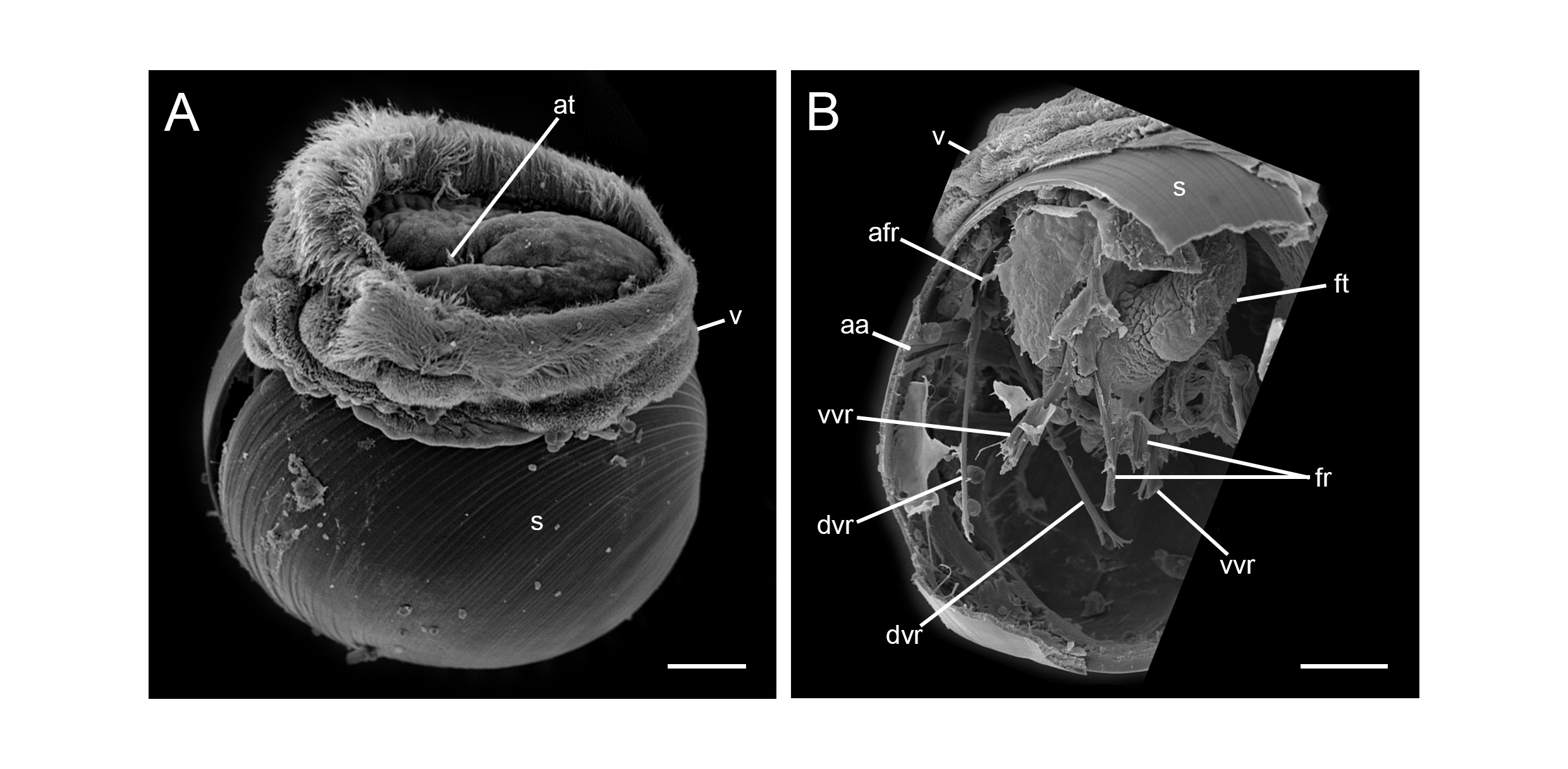

Supplement: Additional file 1: Figure S1. — Scanning electron micrographs of late-stage Lyrodus pedicellatus larvae. [file 12983_2014_90_MOESM1_ESM.jpeg]
