# Supplementary material for: Developmental dynamics of myogenesis in the shipworm Lyrodus pedicellatus (Mollusca: Bivalvia)
Source: Front Zool. 2014 Dec 10;11:90. doi: 10.1186/s12983-014-0090-9 (PMC4282732; doi:10.1186/s12983-014-0090-9)
Supplement: Additional file 2: — Description of the methods used to generate the scanning electron micrograph shown in Supplemental Figure 1. [file 12983_2014_90_MOESM2_ESM.docx]

**Supplemental Information**

**Developmental dynamics of myogenesis in the shipworm *Lyrodus pedicellatus* (Mollusca: Bivalvia)**

**Andrea Wurzinger-Mayer, J. Reuben Shipway, Alen Kristof, Thomas Schwaha, Simon M. Cragg, Andreas Wanninger**

**Inventory of Supplemental Information:**

**Supplemental Figure S1** shows late larvae - images, generated by SEM

**Supplemental Figure Legend**

**Supplemental Information**

**Supplemental Procedures**

*Narcotization*

Prior to fixation, free swimming larvae were narcotized by addition of 7 % MgCl_2_ solution to the seawater and by rapidly reducing temperature by placing samples on ice. After immobilisation of larvae, fixative was gradually added to seawater for 30 minutes. Specimens were then transferred into fixative for a further 30 minutes.

*Primary Fixation*

Samples were fixed with 4 % v/v glutaraldehyde in a cacodylate buffer consisting of 0.2 M sodium cacodylate, 0.3 M NaCl and 2 mM CaCl_2_. If necessary, pH was adjusted to 7.4 using 0.2 M HCl. Samples were then rinsed (2 × 15 min each) in the 0.2 M cacodylate buffer.

*Postfixation*

All samples were postfixed in 1 % aqueous OsO_4_ in a 0.2 M cacodylate buffer for one hour at room temperature. Samples were then rinsed in a 0.2M cacodylate buffer (2 × 15 min), followed by a final rinse with distilled H_2_O. Specimens were then stored at 4 ºC.

*Dehydration*

Samples were taken through a graded ethanol series of 30 %, 50 %, 70 %, 90 % and absolute ethanol, consisting of two ten minute washes at each grade.

*Drying*

Samples were placed in an open Petri dish, submerged in hexamethyldisilazane (HMDS), and were left overnight in a fume hood at room temperature until the solution had evaporated. All fixation, dehydration and drying procedures were performed at room temperature.

*Mounting*

Using a stereomicroscope, specimens were orientated onto a double-sided carbon sticky tab, which was adhered to an aluminium stub. In the case of larger specimens, carbon cement was used to avoid charging of specimens.

*Coating*

Samples were coated with a gold and palladium target using a Polaron E5000sputter coater (Polaron Equipment Ltd, Hertfordshire, UK)..

*Scanning Electron Microscopy*

Specimens were examined using a JEOL JSM 35C Scanning Electron Microscope (Japan Electron Optical Limited, Milton Keynes, UK) at an accelerating voltage of 15 kV. The resulting images were scanned using an Epson Perfection3170 Photo Scanner at a resolution of 800 dpi, and were saved in the JPEG format.

**Supplemental Figure Legend**

**Figure S1:** Scanning electron micrographs showing late larval stages of *Lyrodus pedicellatus.* Scale bars represent 50 µm. Anterior is upwards in both aspects. A is in an anterolateral view, B is in a ventrolateral view. Ventral is to the left in A, to the right in B. (A) Two hemispherical valves form the round shell (s). Ventrally, folds of the ciliated velum (v) result in a typical lobed appearance. The apical tuft (at) marks the centre of the velum. (B) Part of a pediveliger with partially fractured shells (s) revealing the internal anatomy. Paired dorsal (dvr) and ventral (vvr) velum retractors extend toward the velum (v). The two portions of the anterior adductor (aa) span between the left and right valve. Note the fragment of the accessory foot retractor (afr), the paired foot retractors (fr) and the well-developed foot (ft).
